# Three-Alarm System: Revisited to treat Thumb-sucking Habit

**DOI:** 10.5005/jp-journals-10005-1289

**Published:** 2015-04-28

**Authors:** Raghavendra M Shetty, Manoj Shetty, N Shridhar Shetty, Anushka Deoghare

**Affiliations:** PhD Scholar, Department of Dentistry, AB Shetty Memorial Institute of Dental Sciences, Nitte University, Mangalore, Karnataka, India; PhD Guide, Department of Dentistry, AB Shetty Memorial Institute of Dental Sciences, Nitte University, Mangalore, Karnataka, India; PhD Co-Guide, Department of Dentistry, AB Shetty Memorial Institute of Dental Sciences, Nitte University, Mangalore, Karnataka, India; Postgraduate Student, Department of Pediatric Dentistry, Chhattisgarh Dental College and Research Institute, Rajnandgaon, Chhattisgarh India

**Keywords:** Three-alarm system, Thumb sucking, Finger sucking, RURS’ elbow guard.

## Abstract

Thumb and digit-sucking habits or non-nutritive sucking are considered to be the most prevalent among oral habits. Most children stop thumb sucking on their own. If the habit continues beyond 3 to 4 years of age, it not only affects the dental occlusion, but the shape of the thumb/digit may be altered as well. This article presents the management of thumb sucking by modified RURS, elbow guard incorporated with revised ‘three-alarm’ system.

**How to cite this article:** Shetty RM, Shetty M, Shetty NS, Deoghare A. Three-Alarm System: Revisited to treat Thumb-sucking Habit. Int J Clin Pediatr Dent 2015;8(1):82-86.

## INTRODUCTION

Non-nutritive sucking habit can be considered as first step in the development of child’s self-regulation and ability to control emotions.^1^ Thumb sucking is a form of non-nutritive sucking occurring as early as the 29th week of gestation and is seen commonly in infants and peaks at 18 to 21 months of age.^2^ Thumb/finger sucking habits, or non-nutritive sucking are considered to be the most prevalent of oral habits, with a reported incidence ranging from 13% to almost 100% at some time during infancy.^1,3^ The finger-sucking habit, normal in the first 2 or 3 years of life, may cause permanent damage if continued beyond this age.^4^ The oral habits persists usually due to physical and emotional stimuli, such as boredom, hunger, stress, hyperactivity, pleasure, sadness, and various kinds of disabilities. Increase in the child’s level of stress or anxiety can also account for continuation of the sucking habit.^5^

The two theories about finger/digit sucking involve emotional and learned behavior. The emotional theory is Freudian based and relates finger sucking to the oral phase of child development and if sucking continues beyond the oral phase of child development, it becomes a fixation. Digit/finger sucking at a later stage is usually considered a sign of regression. Fixation and regression are the signs of emotional disturbance. The learned behavior theory stems from an adaptive response and suggests that sucking is an innate urge in infants and that finger sucking is an outlet for an excess sucking urge because of efficient feeding, either bottle feeding or breastfeeding by a nutritionally competent mother. Excess sucking urge is expressed as non-nutritive sucking when feeding is quickly and efficiently satisfied.^5,6^ Learned behavior theory has gained favor recently.^7^

The prevalence of a digit-sucking habit decreases with age, and most children abandon this activity by 3.5 to 4 years of age. On occasion, individuals may continue to exhibit a digit habit throughout childhood and even into the adult years. Prolonged digit sucking habit may affect the occlusion and orofacial skeletal system. Frequency and duration of the habit, intensity of the sucking, relationship of the dental arches, and the child’s state of health are the factors effective in the development of dental and skeletal problems.^5^

Reported maxillary changes associated with a prolonged sucking habit are proclination of the maxillary incisors,^8,9^ increased maxillary arch length,^8^ anterior placement of the maxillary apical base,^8^ increased sella-nasion-point A angle,^9^ and decreased palatal arch width. Effects on the mandible include proclination of the mandibular incisors,^8^ increased intermolar distance and decreased sella-nasion-point B angle. Other dental alterations are increased overjet,^8,10^ decreased overbite,^8,11,12^ and posterior crossbite.^13,14^ The response to the changes in the axial inclination of the incisors is anterior rotation of the occlusal plane. Underlying mechanisms of the mal-occlusion are direct pressure from the digit and reduced intraoral pressure produced by sucking.^8^

Since prolonged finger sucking may cause permanent damage to digits/finger, necessitating corrective surgery, the habit should be stopped at an earlier age, before finger deformity or malocclusion have had time to develop.^9^ Once the decision for treatment has been made, one must next determine what intervention is appropriate. The treatment options that are usually considered are age-appropriate explanations to the child, positive reinforcement, digital reminders, and fixed/removable intra-oral habit breaking appliances to prevent sealing of the digit against the palate and to eliminate the pleasure a ssociated with the habit.^6^ RUR S’ elbow guard was success-fully used to break thumb sucking of the child with Hurler Syndrome,^15^ and even in the child with primary dentition.^16^ Steps in fabrication of RURS’ elbow guard is given in detail in our previous published article.^15^

Norton and Gellin^17^ introduced a ‘three-alarm’ system which is often effective in stopping the thumb sucking habit in the mature child. A chart is designed with the days of the week and blank spaces. During the hours the child usually engages in his habit he is told to wrap whatever digit he sucks in coarse adhesive tape. When he feels this tape in his mouth this is a ‘first alarm’ and reminds him to stop. At the same time elbow of the arm with the offending thumb is firmly but not tightly wrapped in a 2 inch elastic bandage obtainable in any drug store. Safety pins are placed in the proximal and distal ends of the bandage, and one is placed lengthwise at the medial bend of the elbow. When he sucks again the closed pin mildly jabbing indicates a ‘second alarm’ to stop sucking. If the child persists the elastic bandage will tightened and his hand fall asleep as a ‘third and final alarm’. However, this alarm system has some drawbacks, such as the opening up of pin, prick or injury to the elbow by the pin. So the ‘three-alarm system’ was revised by modifying RURS’ elbow guard.

This article presents a case report of a child with thumb sucking habit. A unique appliance to prevent thumb sucking was developed by modifying RURS’ elbow guard and was successfully used to break thumb sucking of the child with revised three-alarm system.

## CASE REPORT

**Fig. 1:**
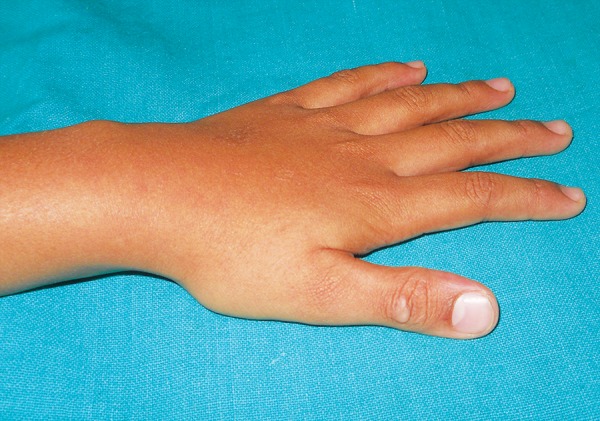
Keratinization and callus formation on the left thumb

**Fig. 2:**
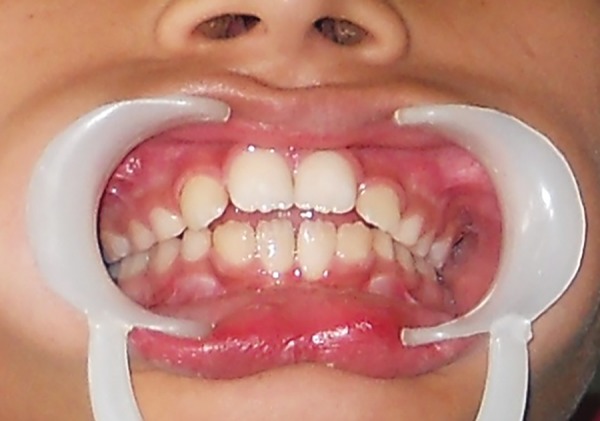
Intraoral view exhibiting mild open bite

A 9 years old child accompanied by his mother reported to the department of Pedodontics and Preventive Dentistry with a chief complaint of thumb sucking. Child’s mother revealed that the child would stop sucking his thumb if reminded, but was not practically possible to monitor all the time as she had another two children to take care who were of 2 and 4 years old. Extraoral examination revealed exceptionally clean and chapped left thumb with keratinisation and callus formation (Fig. 1). Intraoral examination revealed a mild open bite (Fig. 2).

Now specific attention had to be given to prevent thumb sucking to the child where he needed a reminder therapy. It was decided to place a modified RURS’ elbow guard to stop the habit, as it restricted the thumb from reaching the mouth and also would alarm the child not to suck the thumb. An orthopedic surgeon was also consulted before starting the procedure.

**Fig. 3:**
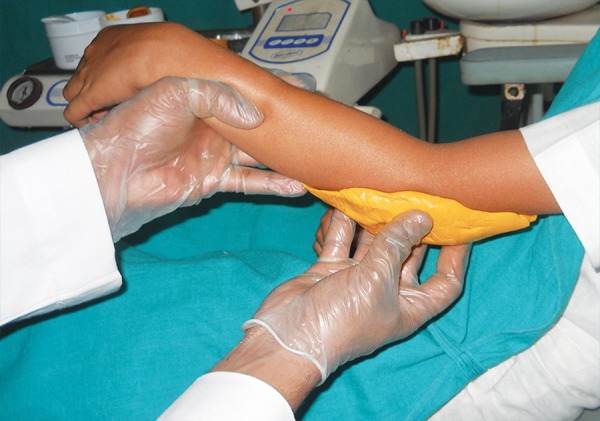
Impression making of the elbow using vinyl polysiloxane putty impression material

**Fig. 4:**
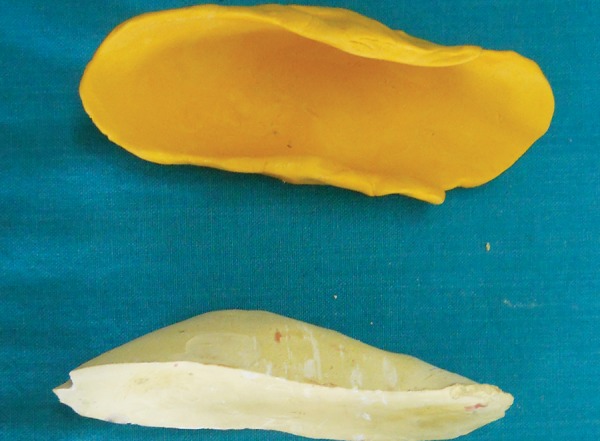
Cast obtained from the impression

**Fig. 5:**
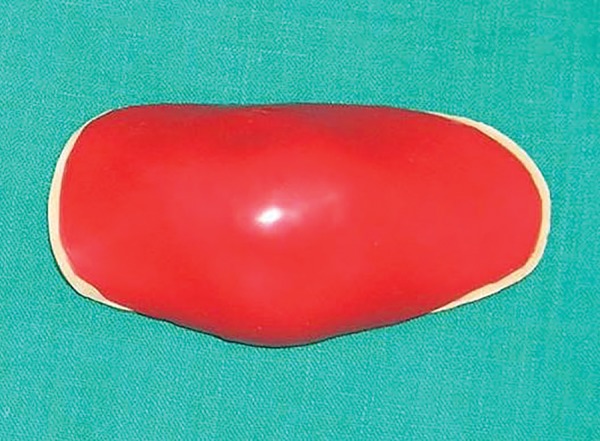
Two layers of modelling wax adapted over the cast as a spacer

**Fig. 6:**
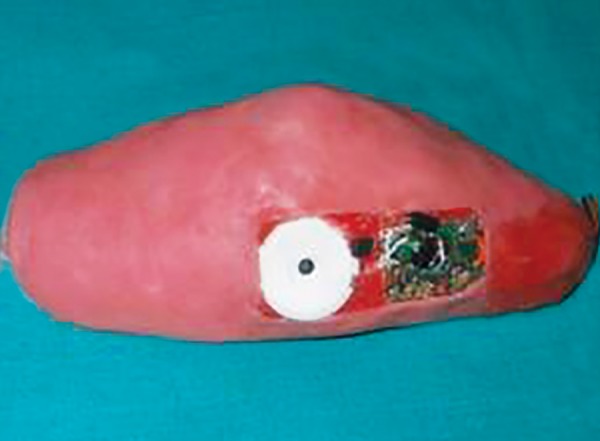
Acrylic elbow guard with musical chip and speaker on the outer surface of acrylic elbow guard

**Fig. 7:**
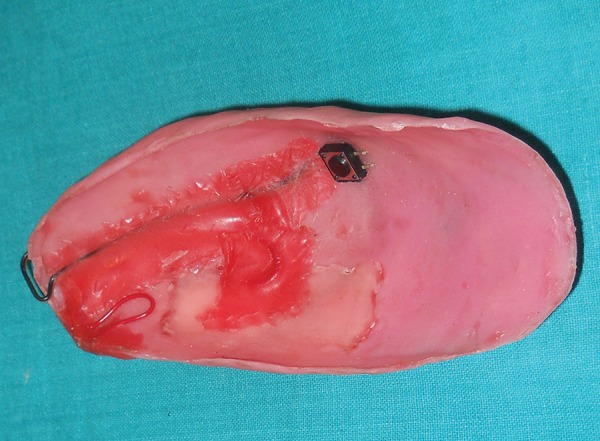
Acrylic elbow guard with switch button on the inner surface of the acrylic elbow guard

**Fig. 8:**
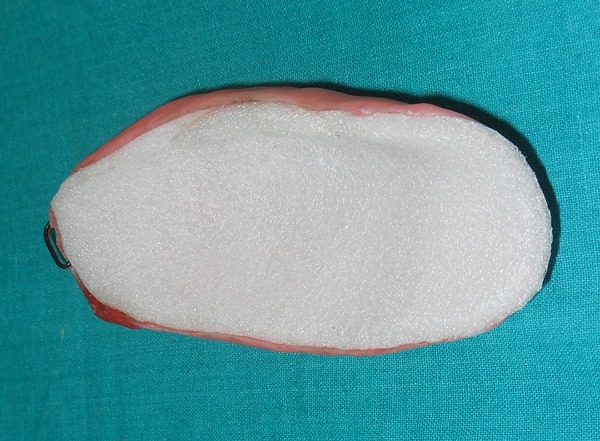
Acrylic elbow guard after placement of a layer of sponge

**Fig. 9:**
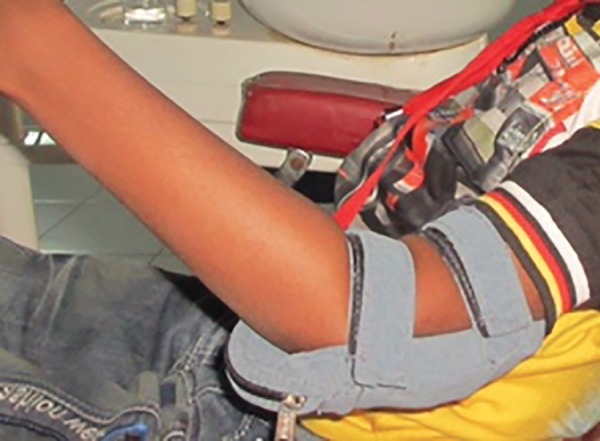
Patient wearing modified RURS’ elbow guard

An impression of the elbow was made (Fig. 3) and a cast was obtained (Fig. 4). Two layers of modeling wax were adapted to the cast which acted as a spacer (Fig. 5). Acrylisation was done using self cure acrylic. A musical chip with speaker was incorporated on the outer side of the acrylic elbow guard during acrylisation (Fig. 6). Spacer was removed and the switch button was placed in the inner side of the acrylic elbow guard. (Fig. 7) and was covered with a layer of sponge (Fig. 8) for cushioning and to allow limited movements of the elbow. A envelop type cover with a zip and velcro strap was stitched over the acrylic elbow guard and delivered to the child (Fig. 9). So, whenever, the child tries to suck the thumb or digit the switch button was pressed by the elbow joint and music would play reminding the child to stop the habit.

When the patient returned for follow-up after 1 month, it was observed that the skin of the thumb was healing. His parents mentioned that the child easily adapted to the appliance. He was recalled for follow-up at 1 month intervals, and he used the appliance for 5 to 6 months continuously. The appliance was removed at the end of 6 month when his mother mentioned that the habit was broken.

## DISCUSSION

Every effort should be made to treat finger/digit sucking if the habit is prolonged, because a finger/digit sucking habit that is not broken will result in not only serious deformities and injuries of the digit but also dental malocclu-sions, such as proclination, anterior open bite, flared maxillary anterior teeth leading to increased overjet and retruded mandibular incisors, posterior crossbite due to transverse maxillary deficiency, and chances of class II malocclusion. The swallowing pattern and speech of the child also may be affected.^12,14,19^ Several methods have been described for the treatment of finger-sucking habits in the literature; these methods can be classified as (a) preventive therapy and (b) appliance therapy. Preventive methods include the application of adhesive tape or bitter solution and or wearing a sock, mitten, gloves, thumb guard, long-sleeve gown. Appliance therapy includes the use of fixed or removable habit breakers desig ned to make the sucking habit difficult or unpleasant.^18^ Explanations appropriate to the age of the child and positive reinforcement are other treatment options for digit suckers and are also necessary for the success of clinical management.^20^

Some of the methods discussed above have certain disadvantages. Clinical experiences have shown that a bitter solution usually has a limited effect.^18^ Adhesive tapes may cause infection, sweating and may also reduce the blood circulation,^21^ while the stuff worn on the hand can easily be removed involuntarily during sleep. Use of altered child’s pajamas to prevent the movement of hand to mouth usually increases the child’s frustration and wakefulness;^22^ also, the pajamas method can be used only if the habit is performed during sleep. Fixed orthodontic habit breaking appliance can cause decalcification of enamel surfaces making them more susceptible to caries and gingival Inflammation may also occur; additionally removable appliances need patient cooperation. Intraoral habit breaking appliances can also cause deviation in speech and pronunciation.^18^

Though ‘three-alarm’ system introduced by Norton and Gellin^17^ is often effective but with some drawbacks, such as the opening up of pin, prick or injury to the elbow by the pin. In the case presented, it was decided to revise the three-alarm principle by modifying the RURS’ elbow guard which was safer to the child. The difference in the ‘three-alarm’ system by Norton and Gellin^17^ and the revised three-alarm system is illustrated (Table 1).

**Table 1:** **Table 1:** Difference between the previous and revised three-alarm system

*Alarm*		*Previous three-alarm system (Norton and Gellin, 1968)*		*Revised three-alarm system*	
First		The child feels the tape in his mouth		The child wearing the elbow guard	
Second		The closed pin mildly jabbing the elbow		The music/vibration/siren/recorded voice played when tried to bend the elbow	
Third/final		The bandage is tightened		The elbow guard restricting thumb/finger reaching the mouth	

Fixed intraoral appliance may create difficulties while eating and mentally disabled children may try to remove their appliance and may frequently break it. On the other hand, removable appliances require patient compliance, making it impossible for children with mental retarda-tion.^7^ Applying an appliance on the patient’s elbow in the presented case has some advantages over orthodontic habit breakers as it does not create difficulties during speech and chewing. Above all, general anesthesia is not required to make the impression of the elbow.

Revised three-alarm principle has an advantage over previous one as there is no danger of injury of the pin which is used as a reminder. However, preparation of the modified RURS’ elbow guard appliance is not so simple, but attracts the child to wear it and unlike intraoral habit breakers, this type of appliance does not affect oral hygiene negatively.

The chip may be incorporated with the favorite music such that the child may indulge in hearing the music and stop the thumb/digit sucking habit. However, at school the music may be a disturbance, so the vibration chip used in mobile phones can be used so that vibrations in the elbow guard can remind the child not to suck the thumb/digit. A siren which a child is scared off also can be incorporated to remind or alarm the child to stop the habit.

Clinical observations revealed that the patient accepted the appliance easily. They found it fashionable like a sports wear, so they did not try to remove it. The elbow guard was firm enough to prevent the child from removing the appliance and was also loose enough to allow limited movement and did not interfere with the blood flow and also the finger was protected from the harmful effects of biting. The child abandoned the habit in a short time since the appliance prevented the pleasure of sucking and, interestingly, the music reminded him to stop the habit and was distracted from sucking pleasure.

## CONCLUSION

Revised ‘three-alarm’ system incorporated in RURS’ elbow guard can be an easy way to manage thumb/digit sucking habit. It is an alternative to intraoral habit breakers; this type of extraoral appliance should be preferred because of its advantages. Further studies on a sufficient number of children are required to evaluate the short- and long-term effects of the presented method.
